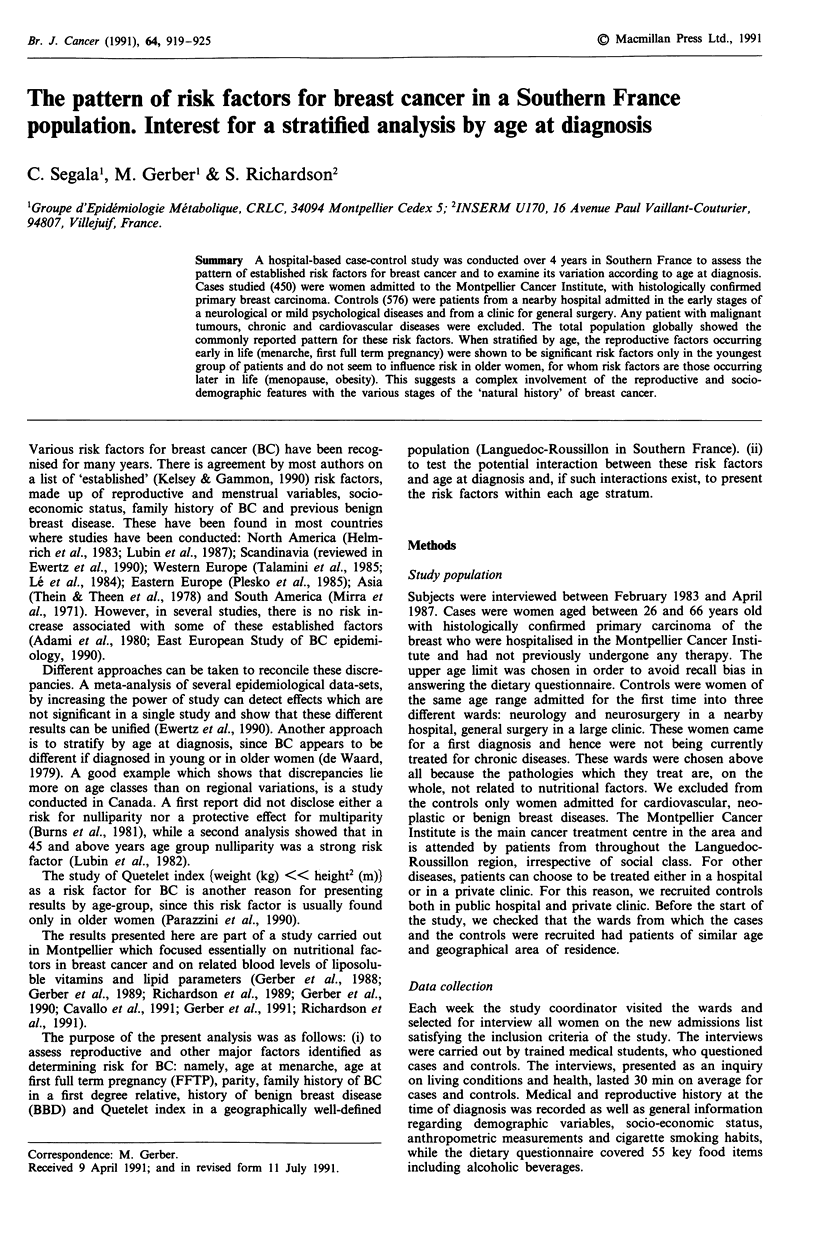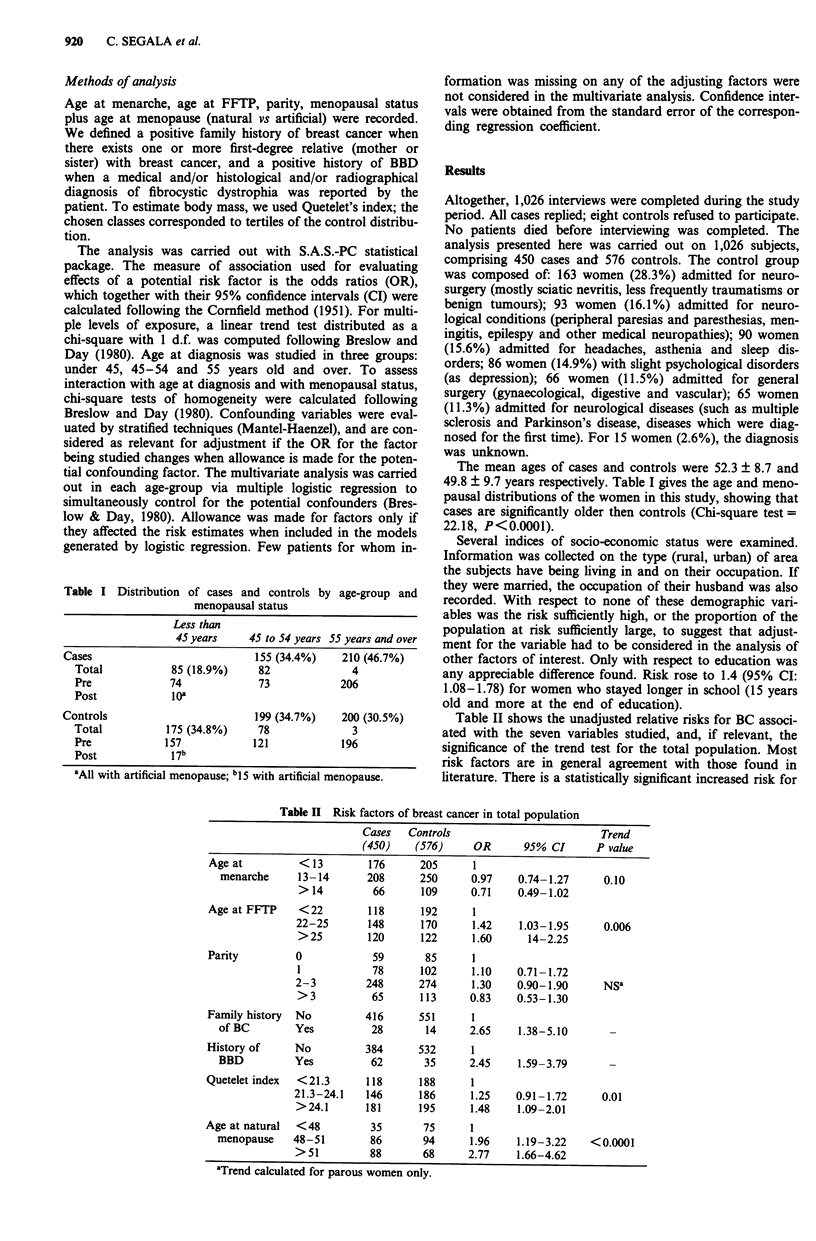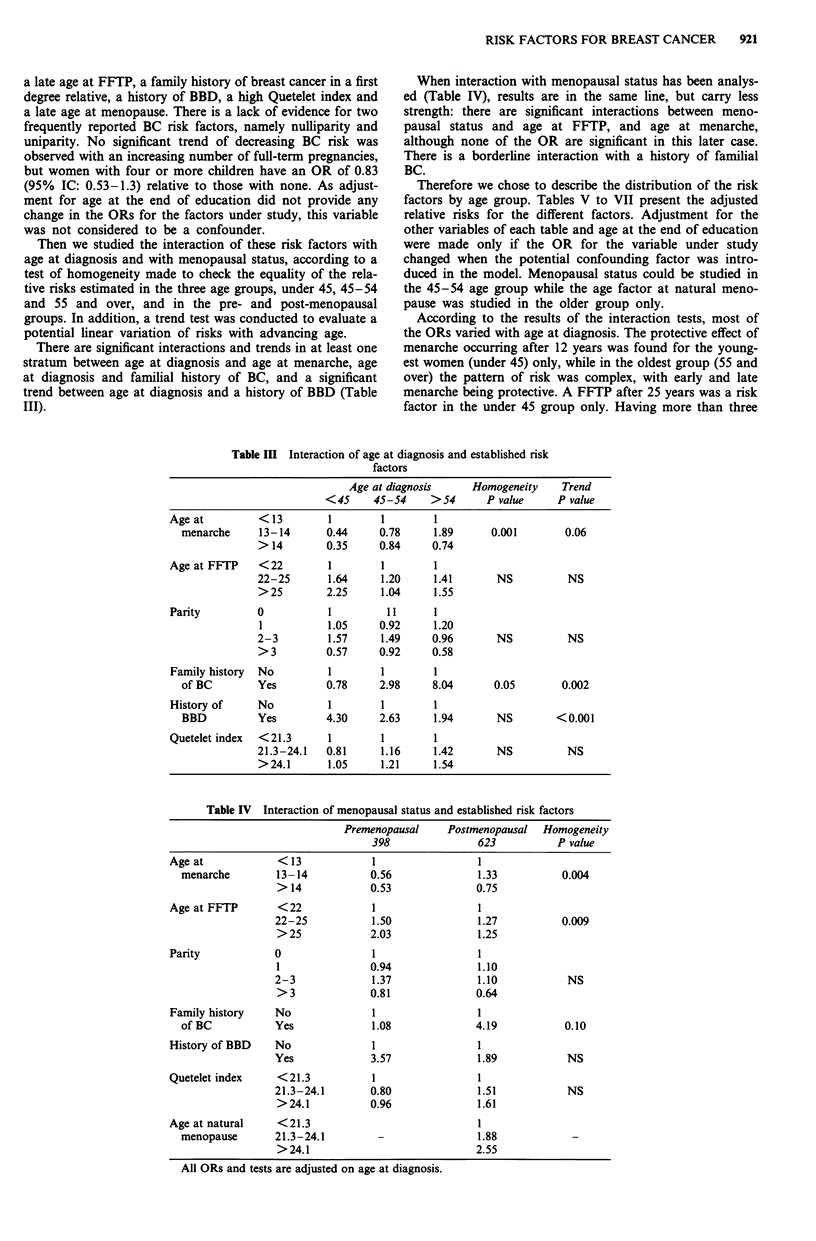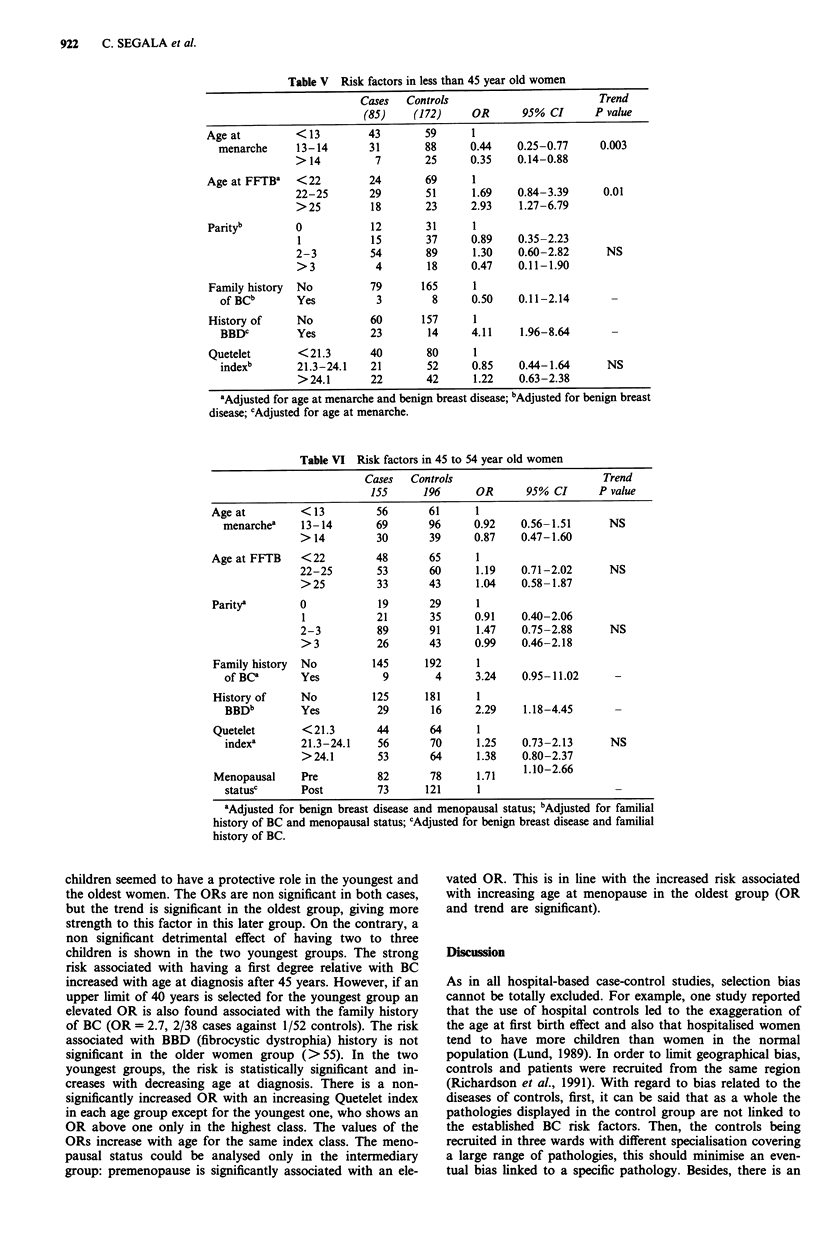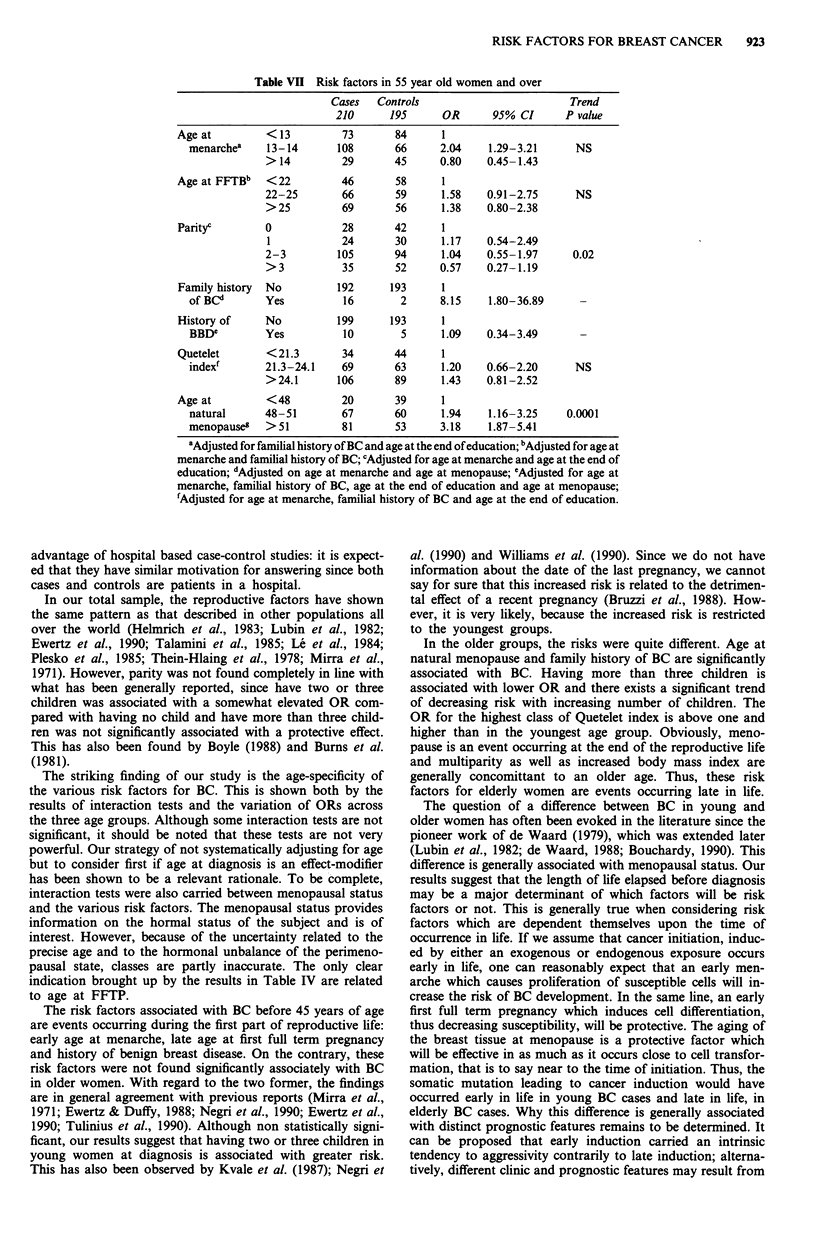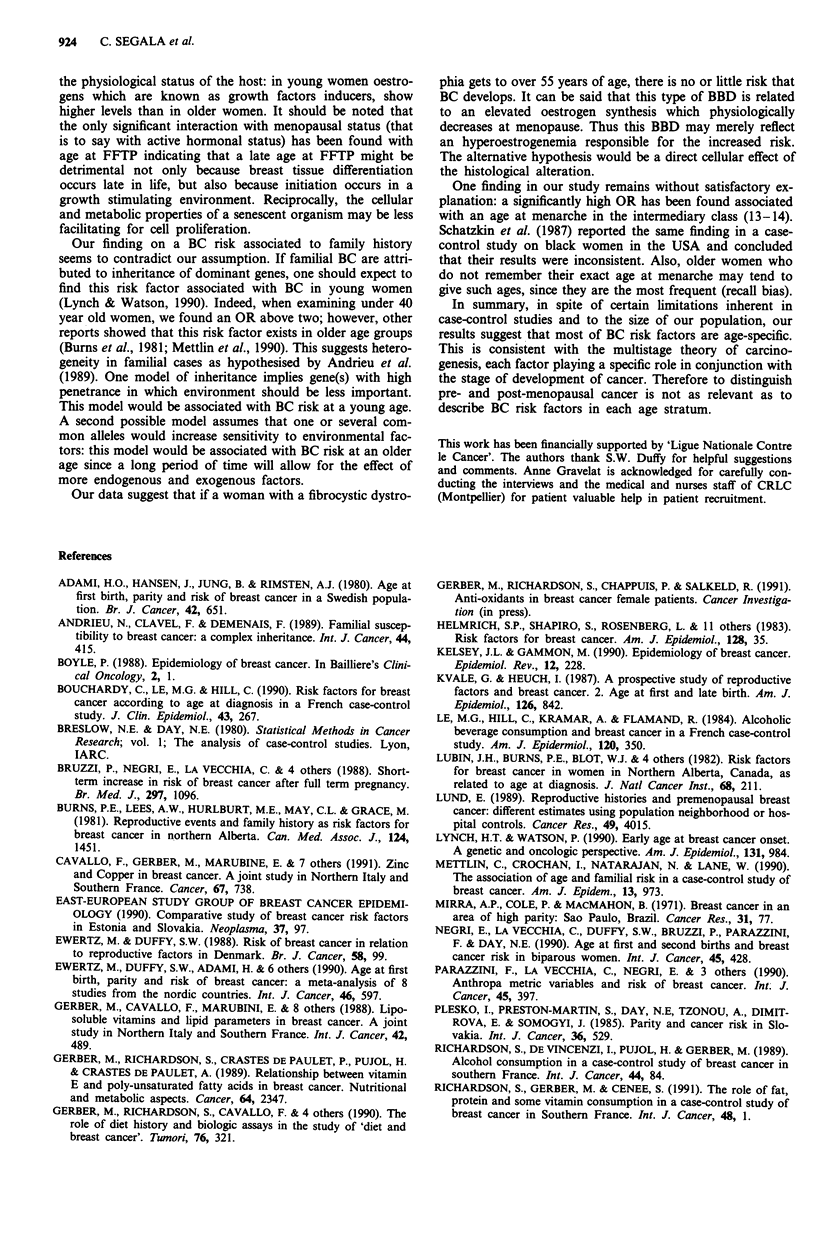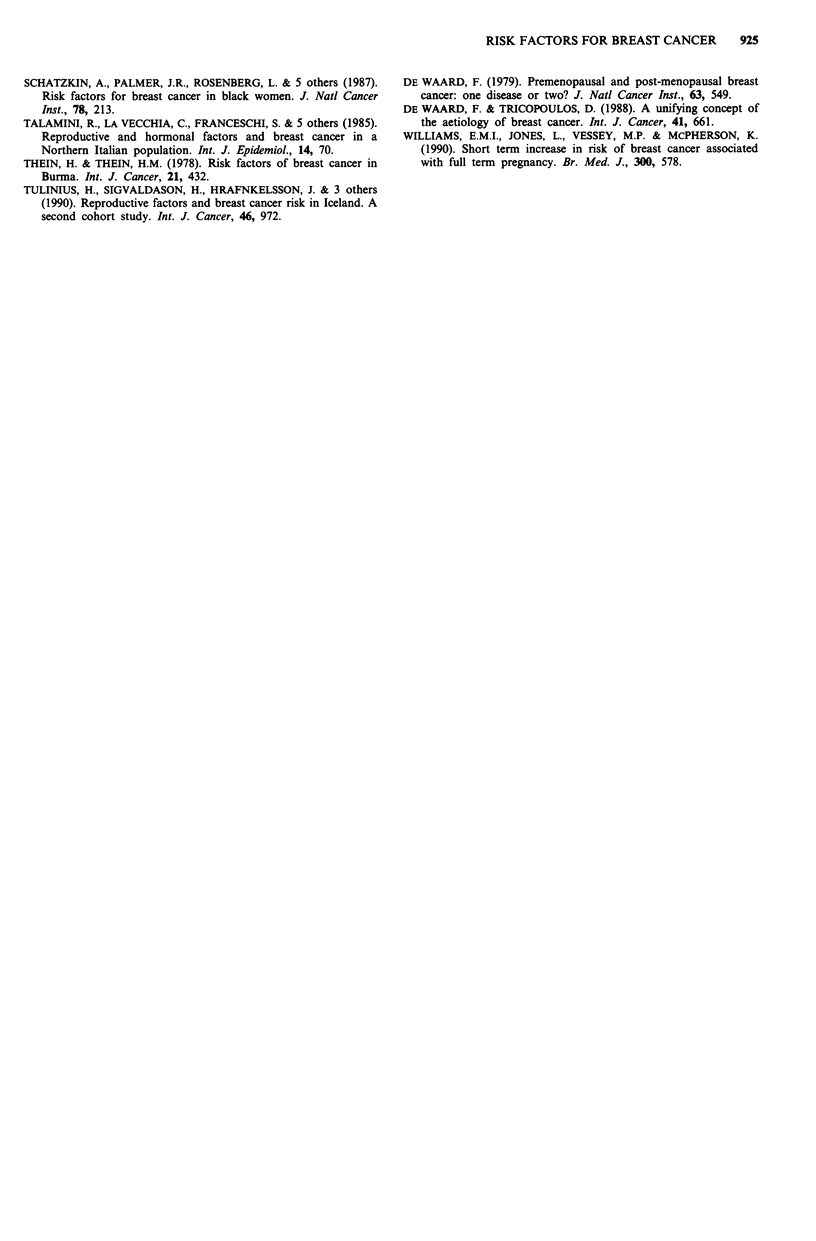# The pattern of risk factors for breast cancer in a southern France population. Interest for a stratified analysis by age at diagnosis.

**DOI:** 10.1038/bjc.1991.427

**Published:** 1991-11

**Authors:** C. Segala, M. Gerber, S. Richardson

**Affiliations:** Groupe d'Epidémiologie Métabolique, CRLC, Montpellier, France.

## Abstract

A hospital-based case-control study was conducted over 4 years in Southern France to assess the pattern of established risk factors for breast cancer and to examine its variation according to age at diagnosis. Cases studied (450) were women admitted to the Montpellier Cancer Institute, with histologically confirmed primary breast carcinoma. Controls (576) were patients from a nearby hospital admitted in the early stages of a neurological or mild psychological diseases and from a clinic for general surgery. Any patient with malignant tumours, chronic and cardiovascular diseases were excluded. The total population globally showed the commonly reported pattern for these risk factors. When stratified by age, the reproductive factors occurring early in life (menarche, first full term pregnancy) were shown to be significant risk factors only in the youngest group of patients and do not seem to influence risk in older women, for whom risk factors are those occurring later in life (menopause, obesity). This suggests a complex involvement of the reproductive and sociodemographic features with the various stages of the 'natural history' of breast cancer.


*Br. J. Cancer* (1991), **64**, 919–925 © Macmillan Press Ltd., 1991

### The pattern of risk factors for breast cancer in a Southern France population. Interest for a stratified analysis by age at diagnosis

C. Segala[1], M. Gerber[1] & S. Richardson[2]

[1]*Groupe d'Epidémiologie Métabolique, CRLC, 34094 Montpellier Cedex 5;* [2]*INSERM U170, 16 Avenue Paul Vaillant-Couturier, 94807, Villejuif, France.*

**Summary** A hospital-based case-control study was conducted over 4 years in Southern France to assess the pattern of established risk factors for breast cancer and to examine its variation according to age at diagnosis. Cases studied (450) were women admitted to the Montpellier Cancer Institute, with histologically confirmed primary breast carcinoma. Controls (576) were patients from a nearby hospital admitted in the early stages of a neurological or mild psychological diseases and from a clinic for general surgery. Any patient with malignant tumours, chronic and cardiovascular diseases were excluded. The total population globally showed the commonly reported pattern for these risk factors. When stratified by age, the reproductive factors occurring early in life (menarche, first full term pregnancy) were shown to be significant risk factors only in the youngest group of patients and do not seem to influence risk in older women, for whom risk factors are those occurring later in life (menopause, obesity). This suggests a complex involvement of the reproductive and socio-demographic features with the various stages of the 'natural history' of breast cancer.

Various risk factors for breast cancer (BC) have been recognised for many years. There is agreement by most authors on a list of 'established' (Kelsey & Gammon, 1990) risk factors, made up of reproductive and menstrual variables, socio-economic status, family history of BC and previous benign breast disease. These have been found in most countries where studies have been conducted: North America (Helmrich *et al.*, 1983; Lubin *et al.*, 1987); Scandinavia (reviewed in Ewertz *et al.*, 1990); Western Europe (Talamini *et al.*, 1985; Lé *et al.*, 1984); Eastern Europe (Plesko *et al.*, 1985); Asia (Thein & Theen *et al.*, 1978) and South America (Mirra *et al.*, 1971). However, in several studies, there is no risk increase associated with some of these established factors (Adami *et al.*, 1980; East European Study of BC epidemiology, 1990).

Different approaches can be taken to reconcile these discrepancies. A meta-analysis of several epidemiological data-sets, by increasing the power of study can detect effects which are not significant in a single study and show that these different results can be unified (Ewertz *et al.*, 1990). Another approach is to stratify by age at diagnosis, since BC appears to be different if diagnosed in young or in older women (de Waard, 1979). A good example which shows that discrepancies lie more on age classes than on regional variations, is a study conducted in Canada. A first report did not disclose either a risk for nulliparity nor a protective effect for multiparity (Burns *et al.*, 1981), while a second analysis showed that in 45 and above years age group nulliparity was a strong risk factor (Lubin *et al.*, 1982).

The study of Quetelet index {weight (kg) << height$^2$ (m)} as a risk factor for BC is another reason for presenting results by age-group, since this risk factor is usually found only in older women (Parazzini *et al.*, 1990).

The results presented here are part of a study carried out in Montpellier which focused essentially on nutritional factors in breast cancer and on related blood levels of liposoluble vitamins and lipid parameters (Gerber *et al.*, 1988; Gerber *et al.*, 1989; Richardson *et al.*, 1989; Gerber *et al.*, 1990; Cavallo *et al.*, 1991; Gerber *et al.*, 1991; Richardson *et al.*, 1991).

The purpose of the present analysis was as follows: (i) to assess reproductive and other major factors identified as determining risk for BC: namely, age at menarche, age at first full term pregnancy (FFTP), parity, family history of BC in a first degree relative, history of benign breast disease (BBD) and Quetelet index in a geographically well-defined population (Languedoc-Roussillon in Southern France). (ii) to test the potential interaction between these risk factors and age at diagnosis and, if such interactions exist, to present the risk factors within each age stratum.

### Methods

### *Study population*

Subjects were interviewed between February 1983 and April 1987. Cases were women aged between 26 and 66 years old with histologically confirmed primary carcinoma of the breast who were hospitalised in the Montpellier Cancer Institute and had not previously undergone any therapy. The upper age limit was chosen in order to avoid recall bias in answering the dietary questionnaire. Controls were women of the same age range admitted for the first time into three different wards: neurology and neurosurgery in a nearby hospital, general surgery in a large clinic. These women came for a first diagnosis and hence were not being currently treated for chronic diseases. These wards were chosen above all because the pathologies which they treat are, on the whole, not related to nutritional factors. We excluded from the controls only women admitted for cardiovascular, neoplastic or benign breast diseases. The Montpellier Cancer Institute is the main cancer treatment centre in the area and is attended by patients from throughout the Languedoc-Roussillon region, irrespective of social class. For other diseases, patients can choose to be treated either in a hospital or in a private clinic. For this reason, we recruited controls both in public hospital and private clinic. Before the start of the study, we checked that the wards from which the cases and the controls were recruited had patients of similar age and geographical area of residence.

### *Data collection*

Each week the study coordinator visited the wards and selected for interview all women on the new admissions list satisfying the inclusion criteria of the study. The interviews were carried out by trained medical students, who questioned cases and controls. The interviews, presented as an inquiry on living conditions and health, lasted 30 min on average for cases and controls. Medical and reproductive history at the time of diagnosis was recorded as well as general information regarding demographic variables, socio-economic status, anthropometric measurements and cigarette smoking habits, while the dietary questionnaire covered 55 key food items including alcoholic beverages.

Correspondence: M. Gerber.
Received 9 April 1991; and in revised form 11 July 1991.

### *Methods of analysis*

Age at menarche, age at FFTP, parity, menopausal status plus age at menopause (natural *vs* artificial) were recorded. We defined a positive family history of breast cancer when there exists one or more first-degree relative (mother or sister) with breast cancer, and a positive history of BBD when a medical and/or histological and/or radiographical diagnosis of fibrocystic dystrophia was reported by the patient. To estimate body mass, we used Quetelet's index; the chosen classes corresponded to tertiles of the control distribution.

The analysis was carried out with S.A.S.-PC statistical package. The measure of association used for evaluating effects of a potential risk factor is the odds ratios (OR), which together with their 95% confidence intervals (CI) were calculated following the Cornfield method (1951). For multiple levels of exposure, a linear trend test distributed as a chi-square with 1 d.f. was computed following Breslow and Day (1980). Age at diagnosis was studied in three groups: under 45, 45–54 and 55 years old and over. To assess interaction with age at diagnosis and with menopausal status, chi-square tests of homogeneity were calculated following Breslow and Day (1980). Confounding variables were evaluated by stratified techniques (Mantel-Haenzel), and are considered as relevant for adjustment if the OR for the factor being studied changes when allowance is made for the potential confounding factor. The multivariate analysis was carried out in each age-group via multiple logistic regression to simultaneously control for the potential confounders (Breslow & Day, 1980). Allowance was made for factors only if they affected the risk estimates when included in the models generated by logistic regression. Few patients for whom information was missing on any of the adjusting factors were not considered in the multivariate analysis. Confidence intervals were obtained from the standard error of the corresponding regression coefficient.

### Results

Altogether, 1,026 interviews were completed during the study period. All cases replied; eight controls refused to participate. No patients died before interviewing was completed. The analysis presented here was carried out on 1,026 subjects, comprising 450 cases and 576 controls. The control group was composed of: 163 women (28.3%) admitted for neurosurgery (mostly sciatic nevritis, less frequently traumatisms or benign tumours); 93 women (16.1%) admitted for neurological conditions (peripheral paresias and paresthesias, meningitis, epilespy and other medical neuropathies); 90 women (15.6%) admitted for headaches, asthenia and sleep disorders; 86 women (14.9%) with slight psychological disorders (as depression); 66 women (11.5%) admitted for general surgery (gynaecological, digestive and vascular); 65 women (11.3%) admitted for neurological diseases (such as multiple sclerosis and Parkinson's disease, diseases which were diagnosed for the first time). For 15 women (2.6%), the diagnosis was unknown.

The mean ages of cases and controls were 52.3 ± 8.7 and 49.8 ± 9.7 years respectively. Table I gives the age and menopausal distributions of the women in this study, showing that cases are significantly older then controls (Chi-square test = 22.18, $P<0.0001$).

Several indices of socio-economic status were examined. Information was collected on the type (rural, urban) of area the subjects have being living in and on their occupation. If they were married, the occupation of their husband was also recorded. With respect to none of these demographic variables was the risk sufficiently high, or the proportion of the population at risk sufficiently large, to suggest that adjustment for the variable had to be considered in the analysis of other factors of interest. Only with respect to education was any appreciable difference found. Risk rose to 1.4 (95% CI: 1.08–1.78) for women who stayed longer in school (15 years old and more at the end of education).

Table II shows the unadjusted relative risks for BC associated with the seven variables studied, and, if relevant, the significance of the trend test for the total population. Most risk factors are in general agreement with those found in literature. There is a statistically significant increased risk for

**Table I** Distribution of cases and controls by age-group and menopausal status

| | *Less than 45 years* | *45 to 54 years* | *55 years and over* |
|---|---|---|---|
| Cases | | 155 (34.4%) | 210 (46.7%) |
| Total | 85 (18.9%) | 82 | 4 |
| Pre | 74 | 73 | 206 |
| Post | 10[a] | | |
| Controls | | 199 (34.7%) | 200 (30.5%) |
| Total | 175 (34.8%) | 78 | 3 |
| Pre | 157 | 121 | 196 |
| Post | 17[b] | | |

[a]All with artificial menopause; [b]15 with artificial menopause.

**Table II** Risk factors of breast cancer in total population

| | | *Cases (450)* | *Controls (576)* | *OR* | *95% CI* | *Trend P value* |
|---|---|---|---|---|---|---|
| Age at menarche | <13 | 176 | 205 | 1 | | |
| | 13–14 | 208 | 250 | 0.97 | 0.74–1.27 | 0.10 |
| | >14 | 66 | 109 | 0.71 | 0.49–1.02 | |
| Age at FFTP | <22 | 118 | 192 | 1 | | |
| | 22–25 | 148 | 170 | 1.42 | 1.03–1.95 | 0.006 |
| | >25 | 120 | 122 | 1.60 | 14–2.25 | |
| Parity | 0 | 59 | 85 | 1 | | |
| | 1 | 78 | 102 | 1.10 | 0.71–1.72 | |
| | 2–3 | 248 | 274 | 1.30 | 0.90–1.90 | NS[a] |
| | >3 | 65 | 113 | 0.83 | 0.53–1.30 | |
| Family history of BC | No | 416 | 551 | 1 | | |
| | Yes | 28 | 14 | 2.65 | 1.38–5.10 | – |
| History of BBD | No | 384 | 532 | 1 | | |
| | Yes | 62 | 35 | 2.45 | 1.59–3.79 | – |
| Quetelet index | <21.3 | 118 | 188 | 1 | | |
| | 21.3–24.1 | 146 | 186 | 1.25 | 0.91–1.72 | 0.01 |
| | >24.1 | 181 | 195 | 1.48 | 1.09–2.01 | |
| Age at natural menopause | <48 | 35 | 75 | 1 | | |
| | 48–51 | 86 | 94 | 1.96 | 1.19–3.22 | <0.0001 |
| | >51 | 88 | 68 | 2.77 | 1.66–4.62 | |

[a]Trend calculated for parous women only.

a late age at FFTP, a family history of breast cancer in a first degree relative, a history of BBD, a high Quetelet index and a late age at menopause. There is a lack of evidence for two frequently reported BC risk factors, namely nulliparity and uniparity. No significant trend of decreasing BC risk was observed with an increasing number of full-term pregnancies, but women with four or more children have an OR of 0.83 (95% IC: 0.53–1.3) relative to those with none. As adjustment for age at the end of education did not provide any change in the ORs for the factors under study, this variable was not considered to be a confounder.

Then we studied the interaction of these risk factors with age at diagnosis and with menopausal status, according to a test of homogeneity made to check the equality of the relative risks estimated in the three age groups, under 45, 45–54 and 55 and over, and in the pre- and post-menopausal groups. In addition, a trend test was conducted to evaluate a potential linear variation of risks with advancing age.

There are significant interactions and trends in at least one stratum between age at diagnosis and age at menarche, age at diagnosis and familial history of BC, and a significant trend between age at diagnosis and a history of BBD (Table III).

When interaction with menopausal status has been analysed (Table IV), results are in the same line, but carry less strength: there are significant interactions between menopausal status and age at FFTP, and age at menarche, although none of the OR are significant in this later case. There is a borderline interaction with a history of familial BC.

Therefore we chose to describe the distribution of the risk factors by age group. Tables V to VII present the adjusted relative risks for the different factors. Adjustment for the other variables of each table and age at the end of education were made only if the OR for the variable under study changed when the potential confounding factor was introduced in the model. Menopausal status could be studied in the 45–54 age group while the age factor at natural menopause was studied in the older group only.

According to the results of the interaction tests, most of the ORs varied with age at diagnosis. The protective effect of menarche occurring after 12 years was found for the youngest women (under 45) only, while in the oldest group (55 and over) the pattern of risk was complex, with early and late menarche being protective. A FFTP after 25 years was a risk factor in the under 45 group only. Having more than three

**Table III** Interaction of age at diagnosis and established risk factors

| | | *Age at diagnosis* | | | *Homogeneity* | *Trend* |
|---|---|---|---|---|---|---|
| | | *<45* | *45–54* | *>54* | *P value* | *P value* |
| Age at menarche | <13 | 1 | 1 | 1 | | |
| | 13–14 | 0.44 | 0.78 | 1.89 | 0.001 | 0.06 |
| | >14 | 0.35 | 0.84 | 0.74 | | |
| Age at FFTP | <22 | 1 | 1 | 1 | | |
| | 22–25 | 1.64 | 1.20 | 1.41 | NS | NS |
| | >25 | 2.25 | 1.04 | 1.55 | | |
| Parity | 0 | 1 | 11 | 1 | | |
| | 1 | 1.05 | 0.92 | 1.20 | | |
| | 2–3 | 1.57 | 1.49 | 0.96 | NS | NS |
| | >3 | 0.57 | 0.92 | 0.58 | | |
| Family history of BC | No | 1 | 1 | 1 | | |
| | Yes | 0.78 | 2.98 | 8.04 | 0.05 | 0.002 |
| History of BBD | No | 1 | 1 | 1 | | |
| | Yes | 4.30 | 2.63 | 1.94 | NS | <0.001 |
| Quetelet index | <21.3 | 1 | 1 | 1 | | |
| | 21.3–24.1 | 0.81 | 1.16 | 1.42 | NS | NS |
| | >24.1 | 1.05 | 1.21 | 1.54 | | |

**Table IV** Interaction of menopausal status and established risk factors

| | | *Premenopausal 398* | *Postmenopausal 623* | *Homogeneity P value* |
|---|---|---|---|---|
| Age at menarche | <13 | 1 | 1 | |
| | 13–14 | 0.56 | 1.33 | 0.004 |
| | >14 | 0.53 | 0.75 | |
| Age at FFTP | <22 | 1 | 1 | |
| | 22–25 | 1.50 | 1.27 | 0.009 |
| | >25 | 2.03 | 1.25 | |
| Parity | 0 | 1 | 1 | |
| | 1 | 0.94 | 1.10 | |
| | 2–3 | 1.37 | 1.10 | NS |
| | >3 | 0.81 | 0.64 | |
| Family history of BC | No | 1 | 1 | |
| | Yes | 1.08 | 4.19 | 0.10 |
| History of BBD | No | 1 | 1 | |
| | Yes | 3.57 | 1.89 | NS |
| Quetelet index | <21.3 | 1 | 1 | |
| | 21.3–24.1 | 0.80 | 1.51 | NS |
| | >24.1 | 0.96 | 1.61 | |
| Age at natural menopause | <21.3 | | 1 | |
| | 21.3–24.1 | – | 1.88 | – |
| | >24.1 | | 2.55 | |

All ORs and tests are adjusted on age at diagnosis.

**Table V** Risk factors in less than 45 year old women

| | | *Cases (85)* | *Controls (172)* | *OR* | *95% CI* | *Trend P value* |
|---|---|---|---|---|---|---|
| Age at menarche | <13 | 43 | 59 | 1 | | |
| | 13–14 | 31 | 88 | 0.44 | 0.25–0.77 | 0.003 |
| | >14 | 7 | 25 | 0.35 | 0.14–0.88 | |
| Age at FFTB[a] | <22 | 24 | 69 | 1 | | |
| | 22–25 | 29 | 51 | 1.69 | 0.84–3.39 | 0.01 |
| | >25 | 18 | 23 | 2.93 | 1.27–6.79 | |
| Parity[b] | 0 | 12 | 31 | 1 | | |
| | 1 | 15 | 37 | 0.89 | 0.35–2.23 | |
| | 2–3 | 54 | 89 | 1.30 | 0.60–2.82 | NS |
| | >3 | 4 | 18 | 0.47 | 0.11–1.90 | |
| Family history of BC[b] | No | 79 | 165 | 1 | | |
| | Yes | 3 | 8 | 0.50 | 0.11–2.14 | – |
| History of BBD[c] | No | 60 | 157 | 1 | | |
| | Yes | 23 | 14 | 4.11 | 1.96–8.64 | – |
| Quetelet index[b] | <21.3 | 40 | 80 | 1 | | |
| | 21.3–24.1 | 21 | 52 | 0.85 | 0.44–1.64 | NS |
| | >24.1 | 22 | 42 | 1.22 | 0.63–2.38 | |

[a]Adjusted for age at menarche and benign breast disease; [b]Adjusted for benign breast disease; [c]Adjusted for age at menarche.

**Table VI** Risk factors in 45 to 54 year old women

| | | *Cases 155* | *Controls 196* | *OR* | *95% CI* | *Trend P value* |
|---|---|---|---|---|---|---|
| Age at menarche[a] | <13 | 56 | 61 | 1 | | |
| | 13–14 | 69 | 96 | 0.92 | 0.56–1.51 | NS |
| | >14 | 30 | 39 | 0.87 | 0.47–1.60 | |
| Age at FFTB | <22 | 48 | 65 | 1 | | |
| | 22–25 | 53 | 60 | 1.19 | 0.71–2.02 | NS |
| | >25 | 33 | 43 | 1.04 | 0.58–1.87 | |
| Parity[a] | 0 | 19 | 29 | 1 | | |
| | 1 | 21 | 35 | 0.91 | 0.40–2.06 | |
| | 2–3 | 89 | 91 | 1.47 | 0.75–2.88 | NS |
| | >3 | 26 | 43 | 0.99 | 0.46–2.18 | |
| Family history of BC[a] | No | 145 | 192 | 1 | | |
| | Yes | 9 | 4 | 3.24 | 0.95–11.02 | – |
| History of BBD[b] | No | 125 | 181 | 1 | | |
| | Yes | 29 | 16 | 2.29 | 1.18–4.45 | – |
| Quetelet index[a] | <21.3 | 44 | 64 | 1 | | |
| | 21.3–24.1 | 56 | 70 | 1.25 | 0.73–2.13 | NS |
| | >24.1 | 53 | 64 | 1.38 | 0.80–2.37 | |
| Menopausal status[c] | Pre | 82 | 78 | 1.71 | 1.10–2.66 | |
| | Post | 73 | 121 | 1 | | – |

[a]Adjusted for benign breast disease and menopausal status; [b]Adjusted for familial history of BC and menopausal status; [c]Adjusted for benign breast disease and familial history of BC.

children seemed to have a protective role in the youngest and the oldest women. The ORs are non significant in both cases, but the trend is significant in the oldest group, giving more strength to this factor in this later group. On the contrary, a non significant detrimental effect of having two to three children is shown in the two youngest groups. The strong risk associated with having a first degree relative with BC increased with age at diagnosis after 45 years. However, if an upper limit of 40 years is selected for the youngest group an elevated OR is also found associated with the family history of BC (OR = 2.7, 2/38 cases against 1/52 controls). The risk associated with BBD (fibrocystic dystrophia) history is not significant in the older women group (>55). In the two youngest groups, the risk is statistically significant and increases with decreasing age at diagnosis. There is a non-significantly increased OR with an increasing Quetelet index in each age group except for the youngest one, who shows an OR above one only in the highest class. The values of the ORs increase with age for the same index class. The menopausal status could be analysed only in the intermediary group: premenopause is significantly associated with an elevated OR. This is in line with the increased risk associated with increasing age at menopause in the oldest group (OR and trend are significant).

### Discussion

As in all hospital-based case-control studies, selection bias cannot be totally excluded. For example, one study reported that the use of hospital controls led to the exaggeration of the age at first birth effect and also that hospitalised women tend to have more children than women in the normal population (Lund, 1989). In order to limit geographical bias, controls and patients were recruited from the same region (Richardson *et al.*, 1991). With regard to bias related to the diseases of controls, first, it can be said that as a whole the pathologies displayed in the control group are not linked to the established BC risk factors. Then, the controls being recruited in three wards with different specialisation covering a large range of pathologies, this should minimise an eventual bias linked to a specific pathology. Besides, there is an

**Table VII** Risk factors in 55 year old women and over

| | | *Cases* 210 | *Controls* 195 | *OR* | *95% CI* | *Trend P value* |
|---|---|---|---|---|---|---|
| Age at menarche[a] | <13 | 73 | 84 | 1 | | |
| | 13–14 | 108 | 66 | 2.04 | 1.29–3.21 | NS |
| | >14 | 29 | 45 | 0.80 | 0.45–1.43 | |
| Age at FFTB[b] | <22 | 46 | 58 | 1 | | |
| | 22–25 | 66 | 59 | 1.58 | 0.91–2.75 | NS |
| | >25 | 69 | 56 | 1.38 | 0.80–2.38 | |
| Parity[c] | 0 | 28 | 42 | 1 | | |
| | 1 | 24 | 30 | 1.17 | 0.54–2.49 | |
| | 2–3 | 105 | 94 | 1.04 | 0.55–1.97 | 0.02 |
| | >3 | 35 | 52 | 0.57 | 0.27–1.19 | |
| Family history of BC[d] | No | 192 | 193 | 1 | | |
| | Yes | 16 | 2 | 8.15 | 1.80–36.89 | – |
| History of BBD[e] | No | 199 | 193 | 1 | | |
| | Yes | 10 | 5 | 1.09 | 0.34–3.49 | – |
| Quetelet index[f] | <21.3 | 34 | 44 | 1 | | |
| | 21.3–24.1 | 69 | 63 | 1.20 | 0.66–2.20 | NS |
| | >24.1 | 106 | 89 | 1.43 | 0.81–2.52 | |
| Age at natural menopause[g] | <48 | 20 | 39 | 1 | | |
| | 48–51 | 67 | 60 | 1.94 | 1.16–3.25 | 0.0001 |
| | >51 | 81 | 53 | 3.18 | 1.87–5.41 | |

[a]Adjusted for familial history of BC and age at the end of education; [b]Adjusted for age at menarche and familial history of BC; [c]Adjusted for age at menarche and age at the end of education; [d]Adjusted on age at menarche and age at menopause; [e]Adjusted for age at menarche, familial history of BC, age at the end of education and age at menopause; [f]Adjusted for age at menarche, familial history of BC and age at the end of education.

advantage of hospital based case-control studies: it is expected that they have similar motivation for answering since both cases and controls are patients in a hospital.

In our total sample, the reproductive factors have shown the same pattern as that described in other populations all over the world (Helmrich *et al.*, 1983; Lubin *et al.*, 1982; Ewertz *et al.*, 1990; Talamini *et al.*, 1985; Lê *et al.*, 1984; Plesko *et al.*, 1985; Thein-Hlaing *et al.*, 1978; Mirra *et al.*, 1971). However, parity was not found completely in line with what has been generally reported, since have two or three children was associated with a somewhat elevated OR compared with having no child and have more than three children was not significantly associated with a protective effect. This has also been found by Boyle (1988) and Burns *et al.* (1981).

The striking finding of our study is the age-specificity of the various risk factors for BC. This is shown both by the results of interaction tests and the variation of ORs across the three age groups. Although some interaction tests are not significant, it should be noted that these tests are not very powerful. Our strategy of not systematically adjusting for age but to consider first if age at diagnosis is an effect-modifier has been shown to be a relevant rationale. To be complete, interaction tests were also carried between menopausal status and the various risk factors. The menopausal status provides information on the hormal status of the subject and is of interest. However, because of the uncertainty related to the precise age and to the hormonal unbalance of the perimenopausal state, classes are partly inaccurate. The only clear indication brought up by the results in Table IV are related to age at FFTP.

The risk factors associated with BC before 45 years of age are events occurring during the first part of reproductive life: early age at menarche, late age at first full term pregnancy and history of benign breast disease. On the contrary, these risk factors were not found significantly associately with BC in older women. With regard to the two former, the findings are in general agreement with previous reports (Mirra *et al.*, 1971; Ewertz & Duffy, 1988; Negri *et al.*, 1990; Ewertz *et al.*, 1990; Tulinius *et al.*, 1990). Although non statistically significant, our results suggest that having two or three children in young women at diagnosis is associated with greater risk. This has also been observed by Kvale *et al.* (1987); Negri *et al.* (1990) and Williams *et al.* (1990). Since we do not have information about the date of the last pregnancy, we cannot say for sure that this increased risk is related to the detrimental effect of a recent pregnancy (Bruzzi *et al.*, 1988). However, it is very likely, because the increased risk is restricted to the youngest groups.

In the older groups, the risks were quite different. Age at natural menopause and family history of BC are significantly associated with BC. Having more than three children is associated with lower OR and there exists a significant trend of decreasing risk with increasing number of children. The OR for the highest class of Quetelet index is above one and higher than in the youngest age group. Obviously, menopause is an event occurring at the end of the reproductive life and multiparity as well as increased body mass index are generally concomittant to an older age. Thus, these risk factors for elderly women are events occurring late in life.

The question of a difference between BC in young and older women has often been evoked in the literature since the pioneer work of de Waard (1979), which was extended later (Lubin *et al.*, 1982; de Waard, 1988; Bouchardy, 1990). This difference is generally associated with menopausal status. Our results suggest that the length of life elapsed before diagnosis may be a major determinant of which factors will be risk factors or not. This is generally true when considering risk factors which are dependent themselves upon the time of occurrence in life. If we assume that cancer initiation, induced by either an exogenous or endogenous exposure occurs early in life, one can reasonably expect that an early menarche which causes proliferation of susceptible cells will increase the risk of BC development. In the same line, an early first full term pregnancy which induces cell differentiation, thus decreasing susceptibility, will be protective. The aging of the breast tissue at menopause is a protective factor which will be effective in as much as it occurs close to cell transformation, that is to say near to the time of initiation. Thus, the somatic mutation leading to cancer induction would have occurred early in life in young BC cases and late in life, in elderly BC cases. Why this difference is generally associated with distinct prognostic features remains to be determined. It can be proposed that early induction carried an intrinsic tendency to aggressivity contrarily to late induction; alternatively, different clinic and prognostic features may result from the physiological status of the host: in young women oestrogens which are known as growth factors inducers, show higher levels than in older women. It should be noted that the only significant interaction with menopausal status (that is to say with active hormonal status) has been found with age at FFTP indicating that a late age at FFTP might be detrimental not only because breast tissue differentiation occurs late in life, but also because initiation occurs in a growth stimulating environment. Reciprocally, the cellular and metabolic properties of a senescent organism may be less facilitating for cell proliferation.

Our finding on a BC risk associated to family history seems to contradict our assumption. If familial BC are attributed to inheritance of dominant genes, one should expect to find this risk factor associated with BC in young women (Lynch & Watson, 1990). Indeed, when examining under 40 year old women, we found an OR above two; however, other reports showed that this risk factor exists in older age groups (Burns *et al.*, 1981; Mettlin *et al.*, 1990). This suggests heterogeneity in familial cases as hypothesised by Andrieu *et al.* (1989). One model of inheritance implies gene(s) with high penetrance in which environment should be less important. This model would be associated with BC risk at a young age. A second possible model assumes that one or several common alleles would increase sensitivity to environmental factors: this model would be associated with BC risk at an older age since a long period of time will allow for the effect of more endogenous and exogenous factors.

Our data suggest that if a woman with a fibrocystic dystrophia gets to over 55 years of age, there is no or little risk that BC develops. It can be said that this type of BBD is related to an elevated oestrogen synthesis which physiologically decreases at menopause. Thus this BBD may merely reflect an hyperoestrogenemia responsible for the increased risk. The alternative hypothesis would be a direct cellular effect of the histological alteration.

One finding in our study remains without satisfactory explanation: a significantly high OR has been found associated with an age at menarche in the intermediary class (13–14). Schatzkin *et al.* (1987) reported the same finding in a case-control study on black women in the USA and concluded that their results were inconsistent. Also, older women who do not remember their exact age at menarche may tend to give such ages, since they are the most frequent (recall bias).

In summary, in spite of certain limitations inherent in case-control studies and to the size of our population, our results suggest that most of BC risk factors are age-specific. This is consistent with the multistage theory of carcinogenesis, each factor playing a specific role in conjunction with the stage of development of cancer. Therefore to distinguish pre- and post-menopausal cancer is not as relevant as to describe BC risk factors in each age stratum.

This work has been financially supported by 'Ligue Nationale Contre le Cancer'. The authors thank S.W. Duffy for helpful suggestions and comments. Anne Gravelat is acknowledged for carefully conducting the interviews and the medical and nurses staff of CRLC (Montpellier) for patient valuable help in patient recruitment.

### References

ADAMI, H.O., HANSEN, J., JUNG, B. & RIMSTEN, A.J. (1980). Age at first birth, parity and risk of breast cancer in a Swedish population. *Br. J. Cancer*, **42**, 651.

ANDRIEU, N., CLAVEL, F. & DEMENAIS, F. (1989). Familial susceptibility to breast cancer: a complex inheritance. *Int. J. Cancer*, **44**, 415.

BOYLE, P. (1988). Epidemiology of breast cancer. In Bailliere's *Clinical Oncology*, **2**, 1.

BOUCHARDY, C., LE, M.G. & HILL, C. (1990). Risk factors for breast cancer according to age at diagnosis in a French case-control study. *J. Clin. Epidemiol.*, **43**, 267.

BRESLOW, N.E. & DAY, N.E. (1980). *Statistical Methods in Cancer Research*; vol. 1; The analysis of case-control studies. Lyon, IARC.

BRUZZI, P., NEGRI, E., LA VECCHIA, C. & 4 others (1988). Short-term increase in risk of breast cancer after full term pregnancy. *Br. Med. J.*, **297**, 1096.

BURNS, P.E., LEES, A.W., HURLBURT, M.E., MAY, C.L. & GRACE, M. (1981). Reproductive events and family history as risk factors for breast cancer in northern Alberta. *Can. Med. Assoc. J.*, **124**, 1451.

CAVALLO, F., GERBER, M., MARUBINE, E. & 7 others (1991). Zinc and Copper in breast cancer. A joint study in Northern Italy and Southern France. *Cancer*, **67**, 738.

EAST-EUROPEAN STUDY GROUP OF BREAST CANCER EPIDEMIOLOGY (1990). Comparative study of breast cancer risk factors in Estonia and Slovakia. *Neoplasma*, **37**, 97.

EWERTZ, M. & DUFFY, S.W. (1988). Risk of breast cancer in relation to reproductive factors in Denmark. *Br. J. Cancer*, **58**, 99.

EWERTZ, M., DUFFY, S.W., ADAMI, H. & 6 others (1990). Age at first birth, parity and risk of breast cancer: a meta-analysis of 8 studies from the nordic countries. *Int. J. Cancer*, **46**, 597.

GERBER, M., CAVALLO, F., MARUBINI, E. & 8 others (1988). Liposoluble vitamins and lipid parameters in breast cancer. A joint study in Northern Italy and Southern France. *Int. J. Cancer*, **42**, 489.

GERBER, M., RICHARDSON, S., CRASTES DE PAULET, P., PUJOL, H. & CRASTES DE PAULET, A. (1989). Relationship between vitamin E and poly-unsaturated fatty acids in breast cancer. Nutritional and metabolic aspects. *Cancer*, **64**, 2347.

GERBER, M., RICHARDSON, S., CAVALLO, F. & 4 others (1990). The role of diet history and biologic assays in the study of 'diet and breast cancer'. *Tumori*, **76**, 321.

GERBER, M., RICHARDSON, S., CHAPPUIS, P. & SALKELD, R. (1991). Anti-oxidants in breast cancer female patients. *Cancer Investigation* (in press).

HELMRICH, S.P., SHAPIRO, S., ROSENBERG, L. & 11 others (1983). Risk factors for breast cancer. *Am. J. Epidemiol.*, **128**, 35.

KELSEY, J.L. & GAMMON, M. (1990). Epidemiology of breast cancer. *Epidemiol. Rev.*, **12**, 228.

KVALE, G. & HEUCH, I. (1987). A prospective study of reproductive factors and breast cancer. 2. Age at first and late birth. *Am. J. Epidemiol.*, **126**, 842.

LE, M.G., HILL, C., KRAMAR, A. & FLAMAND, R. (1984). Alcoholic beverage consumption and breast cancer in a French case-control study. *Am. J. Epidermiol.*, **120**, 350.

LUBIN, J.H., BURNS, P.E., BLOT, W.J. & 4 others (1982). Risk factors for breast cancer in women in Northern Alberta, Canada, as related to age at diagnosis. *J. Natl Cancer Inst.*, **68**, 211.

LUND, E. (1989). Reproductive histories and premenopausal breast cancer: different estimates using population neighborhood or hospital controls. *Cancer Res.*, **49**, 4015.

LYNCH, H.T. & WATSON, P. (1990). Early age at breast cancer onset. A genetic and oncologic perspective. *Am. J. Epidemiol.*, **131**, 984.

METTLIN, C., CROCHAN, I., NATARAJAN, N. & LANE, W. (1990). The association of age and familial risk in a case-control study of breast cancer. *Am. J. Epidem.*, **13**, 973.

MIRRA, A.P., COLE, P. & MACMAHON, B. (1971). Breast cancer in an area of high parity: Sao Paulo, Brazil. *Cancer Res.*, **31**, 77.

NEGRI, E., LA VECCHIA, C., DUFFY, S.W., BRUZZI, P., PARAZZINI, F. & DAY, N.E. (1990). Age at first and second births and breast cancer risk in biparous women. *Int. J. Cancer*, **45**, 428.

PARAZZINI, F., LA VECCHIA, C., NEGRI, E. & 3 others (1990). Anthropa metric variables and risk of breast cancer. *Int. J. Cancer*, **45**, 397.

PLESKO, I., PRESTON-MARTIN, S., DAY, N.E, TZONOU, A., DIMITROVA, E. & SOMOGYI, J. (1985). Parity and cancer risk in Slovakia. *Int. J. Cancer*, **36**, 529.

RICHARDSON, S., DE VINCENZI, I., PUJOL, H. & GERBER, M. (1989). Alcohol consumption in a case-control study of breast cancer in southern France. *Int. J. Cancer*, **44**, 84.

RICHARDSON, S., GERBER, M. & CENEE, S. (1991). The role of fat, protein and some vitamin consumption in a case-control study of breast cancer in Southern France. *Int. J. Cancer*, **48**, 1.

SCHATZKIN, A., PALMER, J.R., ROSENBERG, L. & 5 others (1987). Risk factors for breast cancer in black women. *J. Natl Cancer Inst.*, **78**, 213.

TALAMINI, R., LA VECCHIA, C., FRANCESCHI, S. & 5 others (1985). Reproductive and hormonal factors and breast cancer in a Northern Italian population. *Int. J. Epidemiol.*, **14**, 70.

THEIN, H. & THEIN, H.M. (1978). Risk factors of breast cancer in Burma. *Int. J. Cancer*, **21**, 432.

TULINIUS, H., SIGVALDASON, H., HRAFNKELSSON, J. & 3 others (1990). Reproductive factors and breast cancer risk in Iceland. A second cohort study. *Int. J. Cancer*, **46**, 972.

DE WAARD, F. (1979). Premenopausal and post-menopausal breast cancer: one disease or two? *J. Natl Cancer Inst.*, **63**, 549.

DE WAARD, F. & TRICOPOULOS, D. (1988). A unifying concept of the aetiology of breast cancer. *Int. J. Cancer*, **41**, 661.

WILLIAMS, E.M.I., JONES, L., VESSEY, M.P. & MCPHERSON, K. (1990). Short term increase in risk of breast cancer associated with full term pregnancy. *Br. Med. J.*, **300**, 578.